# Evaluation of the dietary patterns of individuals with type 1 diabetes who use carbohydrate counting at a public health unit in Brasília-DF

**DOI:** 10.1186/1758-5996-7-S1-A109

**Published:** 2015-11-11

**Authors:** Denise Glória Silva de Paula da Costa, Maria Aparecida Barbosa Nascimento

**Affiliations:** 1 SESDF, Brasília, Brazil

## Objectives

To evaluate the eating behavior of individuals with type 1 diabetes (T1D) and the risk of developing chronic complications.

## Materials and methods

An analytical cross-sectional study with a convenience sample consisted of 23 individuals with type 1 diabetes, who needed Intensive insulin therapy and were treated at a public health unit in which carbohydrate counting was used in the treatment. Through interviews, information on socioeconomic data and feeding behavior was obtained. Anthropometric measurements (weight, height and waist circumference) were also taken.

## Results

It was found that most individuals of the sample correctly applied the carbohydrates counting method. For the majority of the sample, food intake was found adequate when compared with that recommended by the Dietary Guidelines for Brazilian population (Guia Alimentar para a População Brasileira), in regard to the groups of carbohydrates, vegetables, proteins of animal and vegetable origin, oils and fats. Most of the sample had a daily or a 2 to 4 times a week intake of whole foods. In contrast, consumption of dairy products and fruits was insufficient among most participants. It was found that 39% of the sample had some degree of overweight, as measured by the body mass index, and 43.4% of the sample had a waist circumference higher than the recommended value.

## Conclusion

Helping individuals with T1D is essential in maintaining an appropriate body weight and in preventing abdominal obesity. This should be coupled with a balanced diet, which is essential in the treatment in order to minimize long-term complications and aids achieving good glycemic control. It is crucial to offer periodic training to ensure correct application of the carbohydrate counting method.

